# Regulation of methanol utilisation pathway genes in yeasts

**DOI:** 10.1186/1475-2859-5-39

**Published:** 2006-12-14

**Authors:** Franz S Hartner, Anton Glieder

**Affiliations:** 1 Research Centre Applied Biocatalysis GmbH, Petersgasse 14/2, 8010 Graz, Austria; 2 Institute of Molecular Biotechnology, Graz University of Technology, Petersgasse 14/2, 8010 Graz, Austria

## Abstract

Methylotrophic yeasts such as *Candida boidinii*, *Hansenula polymorpha*, *Pichia methanolica *and *Pichia pastoris *are an emerging group of eukaryotic hosts for recombinant protein production with an ever increasing number of applications during the last 30 years. Their applications are linked to the use of strong methanol-inducible promoters derived from genes of the methanol utilisation pathway. These promoters are tightly regulated, highly repressed in presence of non-limiting concentrations of glucose in the medium and strongly induced if methanol is used as carbon source. Several factors involved in this tight control and their regulatory effects have been described so far. This review summarises available data about the regulation of promoters from methanol utilisation pathway genes. Furthermore, the role of *cis *and *trans *acting factors (e.g. transcription factors, glucose processing enzymes) in the expression of methanol utilisation pathway genes is reviewed both in the context of the native cell environment as well as in heterologous hosts.

## Background

The yeast *Saccharomyces cerevisiae *was used by mankind for millennia, mainly in brewing and baking. Because of its unchallenged use for thousands of years, this species, also known as baker's or brewer's yeast, became a synonym for yeast. However, in research and industrial biotechnology more and more yeast species with outstanding characteristics have been investigated, starting in the 1970's to 80's [1-7]. Among them a limited number of yeast species capable of growing on methanol as sole carbon and energy source have been characterised and their application in biotechnology was established for single cell protein production [8] and later for recombinant protein production [9,10]. These so-called methylotrophic yeasts belong to the four genera *Hansenula*, *Pichia*, *Candida *and *Torulopsis *[11-13] including their most prominent representatives *Pichia pastoris*, *Hansenula polymorpha *(*Pichia angusta*), *Candida boidinii *and *Pichia methanolica *[14,15].

The uses of methylotrophic yeasts reported so far have relied on their ability to grow on methanol or at least on the unique regulatory features of their pathways for methanol utilisation. These efforts have been concentrated in three areas:

a) Since the initial reactions of methanol utilisation are compartmentalised within peroxisomes, these organelles are strongly induced upon shifting the cells to methanol as sole carbon source. Therefore these yeasts are frequently used model organisms to study peroxisome biogenesis and function [16-19]. However, methylotrophic yeasts as model organisms in cell biology are not restricted to peroxisome research. *Pichia pastoris *is perfectly suited to study the early secretion pathway because it exhibits a discrete transitional endoplasmic reticulum (ER) and coherent Golgi stacks [20,21].

b) Methylotrophic yeasts or enzymes thereof have been developed for bioconversions or as recognition elements in biosensor applications, mainly for the oxidation or detection of alcohols [22-26].

c) The major application of methylotrophic yeasts is recombinant protein production. As reviewed extensively [27-32], promoter elements derived from genes involved in methanol utilisation, especially the alcohol oxidase promoters, have been utilised to express several hundreds of different proteins in methylotrophic yeasts so far.

Like *Saccharomyces cerevisiae*, methylotrophic yeasts combine the advantages of microorganisms (i.e. simple cultivation on inexpensive growth media, well established genetic techniques and tremendous experience on industrial cultivation and scale-up) with the ability to perform typical eukaryotic protein processing and other posttranslational modifications such as glycosylation. Many more advantages became relevant for pharmaceutical proteins. The products are free of endotoxins and also of oncogenic and viral DNA. Due to their use in food industry for thousands of years some yeasts possess a GRAS status (Generally Regarded As Safe).

The industrial relevance of methylotrophic yeasts is still dramatically increasing due to some technologically interesting features. In contrast to *S. cerevisiae *they belong to the group of Crabtree negative yeasts, thus ethanol production occurs at very low level under fully aerobic conditions. This enables these yeasts to grow to very high cell densities in bioreactor cultures and consequently results in high product yields [6,33]. Secondly, there is a tendency of non-conventional yeasts (including *P. pastoris *and *H. polymorpha*) for secretion of proteins, even with higher molecular mass [2] while in *S. cerevisiae *retention of proteins in the periplasmatic space occurs even for peptides of low molecular weight. The high secretion capacity of methylotrophic yeasts in combination with well-established high cell-density fermentation techniques resulted in secretion of heterologous proteins up to levels of several grams per litre (e.g. phytase [34,35], gelatin [36-40]).

Although hyper-mannosylation occurs less frequently compared to *S. cerevisiae*, the major difference in respect to glycosylation is the lack of hyper-immunogenic terminal α-1,3-linked mannoses in N-linked glycans in proteins produced by *P*.*pastoris *and *H*.*polymorpha *[41-45]. The potential to replace cell culture systems as the preferred production system for therapeutic proteins drives research to engineer methylotrophic yeasts for homogenised and humanised N-linked protein glycosylation. This was already successful in terms of trimming complex Golgi-derived glycan structures to ER-derived core-glycans. Core-glycosylation is conserved among higher and lower eukaryotes [46-48]. Very recently, in an interesting engineering approach the multi-step pathways to synthesise galactose and sialic acid as well as the respective pathways to add these sugars to glycans was introduced into *P. pastoris*, providing a battery of individual strains which add specific and homogenous human-like glycan structures to recombinant proteins [45,49-53]. This breakthrough has opened the door to increasing applications of this methylotrophic yeast for the production of biopharmaceuticals such as therapeutic antibodies, a market so far dominated by *E. coli*, *S. cerevisiae *and mammalian cell lines [53-56].

## Review

### Introduction

The success of methylotrophic yeasts in the production of recombinant proteins is highly linked to the very strong and tightly regulated promoters of some genes of the methanol utilisation pathway (MUT pathway). Many of these genes and their respective promoter regions have been isolated (Table 1). Especially the promoter of the gene coding the first enzyme of the pathway in *Pichia pastoris*, the alcohol oxidase I (*AOX1*), and the corresponding promoters from other methylotrophic yeasts are most commonly applied for recombinant protein production in these hosts [10,29,57]. *H. polymorpha *where the *FMD *(formate dehydrogenase) promoter is most frequently employed is an exception [58,59]. These promoters enabled the production of intracellular as well as secreted proteins up to a concentration of several grams per litre (e.g. [34,35,60]).

**Table 1:** Methanol utilisation pathway genes in methylotrophic yeasts.

Organism^a^	Gene/Promoter	Genbank Acc. No.^b^	Reference^b^
*Candida boidinii *S2 [175]	*AOD1*	M81702	[63]
	*FLD1*	AB085186	[71]
	*FDH1*	AF004096	[70]
	*CTA1*	AB064338	[108]
	*DAS1*	AF086822	[68]

*Hansenula polymorpha *(*Pichia angusta*) CBS4732 (ATCC 34438)	*MOX*	X02425	[84]
	*FLD*	AF364077	[76]
	*FMD*	A06214	[104, 176]
	*DAS*	X02424, A11168	[69, 177]
	*CAT*	X56501	[178]

*Pichia pastoris *NRRL Y-11430 (CBS7435)	*AOX1*	U96967	[62]
	*AOX2*	U96968, X79871	[62]
	*FLD1*	AF066054	[77]
	ZZA1*	S62281	[166]

*Pichia methanolica *IAM12901	*MOD1 *(*AUG1*)	AF141329	[82], [101]
	*MOD2 *(*AUG2*)	AF141330, AB223040	[101]
	*FLD*	AB112935	[78]

^a^Strains used to isolate and characterise genes and promoters.

^b^The Genbank accession numbers (Acc. No.) for the entire gene or the promoter and the respective reference are given. *(from H. Phaff ID 72–1033)

The following chapters review the regulation of these genes and the application of their corresponding promoters for the production of recombinant proteins in methylotrophic and other yeasts.

### The methanol utilisation pathway (MUT pathway)

**Figure 1:**
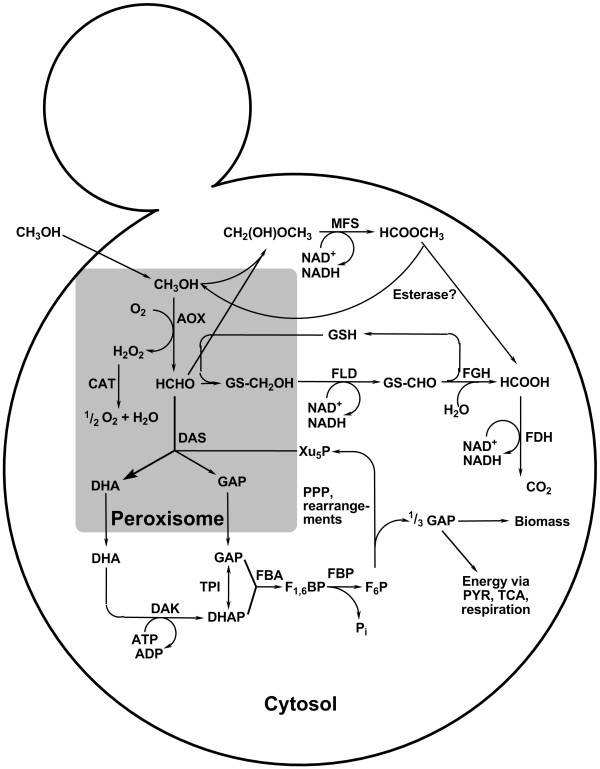
**Methanol utilisation pathway in methylotrophic yeasts: **The main pathways and the respective enzymes working in the methanol metabolism in methylotrophic yeasts are shown. AOX: alcohol oxidase (EC 1.1.3.13), CAT: catalase (EC 1.11.1.6), FLD: formaldehyde dehydrogenase (EC 1.2.1.1), FGH: S-formylglutathione hydrolase (EC 3.1.2.12), FDH: formate dehydrogenase (EC 1.2.1.2), DAS: dihydroxyacetone synthase (EC 2.2.1.3), TPI: triosephosphate isomerase (EC 5.3.1.1), DAK: dihydroxyacetone kinase (EC 2.7.1.29), FBA: fructose 1,6-bisphosphate aldolase (EC 4.1.21.13), FBP: fructose 1,6-bisphosphatase (EC 3.1.3.11), MFS: methylformate synthase; DHA: dihydroxyacetone, GAP: glyceraldehyde 3-phosphate, DHAP: dihydroxyacetone phosphate, F_1,6_BP: fructose 1,6-bisphosphate, F_6_P: fructose 6-phosphate, P_i_: phosphate, Xu_5_P: xylulose 5-phosphate, GSH: glutathione, PYR: pyruvate; PPP: pentose phosphate pathway, TCA: tricarboxylic acid cycle

Methylotrophic yeasts share a unique MUT pathway whose expression is tightly regulated at the level of transcription [61-64]. Since a part of the MUT pathway takes place in the peroxisomes, these organelles proliferate massively upon methanol induction. After induction they can account for up to 80% of the cytoplasmic space [65]. In the first step of methanol utilisation, methanol is oxidised to formaldehyde and hydrogen peroxide by alcohol oxidases (*AOX*, EC 1.1.3.13). The toxic H_2_O_2 _is broken down to oxygen and water by the action of catalase (*CAT*, EC 1.11.1.6). Both enzymes are sequestered in peroxisomes (see Figure 1) [32,66,67]. Formaldehyde is either oxidised by two subsequent dehydrogenase reactions (dissimilation pathway) or assimilated in the cell metabolism by condensation with xylulose 5-phosphate (Xu_5_P). The latter peroxisomal condensation reaction is catalysed by a special transketolase called dihydroxyacetone synthase (*DAS*, 2.2.1.3) [32,64,66,68,69]. *DAS *converts Xu_5_P and formaldehyde into the C_3_-compounds dihydroxyacetone (DHA) and glyceraldehyde 3-phosphate (GAP), which are further metabolised in the cytosol (Figure 1).

In the dissimilation pathway, formaldehyde spontaneously reacts with glutathione to *S*-hydroxymethylglutathione which then is oxidised in two consecutive reactions to carbon dioxide by the action of a glutathione (GSH)- and NAD^+^-dependent formaldehyde dehydrogenase (*FLD*, EC 1.2.1.1) and a NAD^+^-dependent formate dehydrogenase (*FDH *1.2.1.2), both located in the cytosol. NADH, generated in both dehydrogenase reactions, is considered to be used in energy production for growth on methanol [32,70]. Beside the role in energy production, the dissimilation pathway enzymes play a role in the detoxification of formaldehyde and formate, respectively [70,71]. A third enzyme (or enzymatic activity) involved in this pathway, *S*-formylglutatione hydrolase (*FGH*, EC 3.1.2.12) hydrolyses S-formylglutathione to formate and glutathione [71-74]. It participates in the detoxification of formaldehyde and regenerates glutathione [71,72]. While a separate enzyme was found in *C. boidinii*, *Kloeckera *sp. 2201 (a strain of *C. boidinii*) and *P. pastoris*, in *H. polymorpha FGH *activity was found to be associated with the *FDH *[75]. The methanol dissimilation pathway also plays a major role in the metabolism of methylated nitrogen sources such as choline or methylamine where formaldehyde is a by-product. In the latter, detoxification of formaldehyde is the main task of the methanol dissimilation pathway [71,76,77]. Knockout studies on *FLD *and *FDH *strains of *C. boidinii *and *P. methanolica *demonstrated that the *FLD *knockout phenotype is more severe than *FDH *knockout, which was explained by the higher toxicity of formaldehyde compared to formate [71,78].

In general, expression of the methanol pathway genes is repressed by glucose and ethanol and strongly induced by methanol. Ethanol and glucose act via two distinct mechanism to repress the MUT pathway [79-81]. In induced cells the key enzymes alcohol oxidase, dihydroxyacetone synthase and formate dehydrogenase can account for up to 30 % (*AOX*) or 20 % (*DAS*, *FDH*) of the total soluble protein in induced cells [10,14,15]. Due to their role in nitrogen metabolism the regulation of the genes of the dissimilation pathway is slightly different. *AOX *and *DAS *genes are repressed by glucose while methanol is necessary for induction. Induction of *FLD *and *FDH *expression was also accomplished by methylated nitrogen sources such as methylamine or choline, even in the presence of glucose [70,71].

### Alcohol oxidases – the entry into the pathway

The number of genes coding for alcohol oxidases varies among methylotrophic yeasts. While in *Candida boidinii *and *Hansenula polymorpha *only one gene codes for an alcohol oxidase (*AOD1 *and *MOX*, respectively), two genes are found in *Pichia pastoris *and *Pichia methanolica *(*AOX1 *and *AOX2*, and *MOD1 *(*AUG1*) and *MOD2 *(*AUG2*), respectively) [62,63,82-85]. Furthermore, not only is the number of alcohol oxidase genes diverse among the methylotrophic yeasts, but also their regulation in the respective yeasts varies significantly.

The regulation of the *AOX *genes in *C. boidinii *and *H. polymorpha *is very similar: the *HpMOX *and the *CbAOD1 *promoter are repressed during growth on glucose or ethanol as sole carbon source, derepressed in the presence of glycerol and strongly induced by methanol in absence of glucose. The level of derepression on glycerol differs between these two yeasts. While in *C. boidinii *the *AOD1 *derepression on glycerol results in only ~20% activity compared to the methanol-induced level, the *H. polymorpha MOX *activity under these conditions is almost ~80% of the methanol-induced level (Table 2). Expression of *AOD1 *or *MOX *was also repressed when both glucose and methanol were present in the medium (glucose in non-limiting concentrations) while a glycerol and methanol mixture resulted in alcohol oxidase activity which is comparable to the activity in media containing methanol as sole carbon source [57,86-90]. Several other carbon sources (e.g. xylose, ribose and sorbitol) are also able to derepress *MOX *expression in *H. polymorpha*. The expression level on these carbon sources is lower than with glycerol, i.e. only ~50 % (xylose), ~25 % (ribose) or ~10 % (sorbitol) of the fully induced level obtained with methanol [91].

**Table 2:** Repression and derepression effect of carbon sources on *AOX *genes of methylotrophic yeasts.

		Glucose, EtOH	Glycerol	Methanol	Glycerol+MeOH
*P. pastoris *[61, 62, 83, 92, 93]	*AOX1*	Repression	Repression	Induction	Repression
	*AOX2*	Repression	Repression	Induction	Repression
*H. polymorpha *[76, 88–91]	*MOX*	Repression	Derepr. (~60–70%)	Induction	Induction (~100%)
	*FMD*	Repression	Derepr. (~60%)	Induction	
*C. boidinii *[57, 68, 70, 71, 86, 87]	*AOD1*	Repression	Derepr. (~3–30%)	Induction	Induction (~90%)
	*FLD*	No activity	Derepr. (~20%)	Induction	Induction (~70%)
	*FDH*	Repression	No activity	Induction	Induction (~30%)
	*DAS*	Repression	Derepr. (~2%)	Induction	Induction (~70%)
*P. methanolica *[78, 82, 100, 101]	*MOD1*	Repression	Derepr. (~60–70%)	Induction	Induction (~100%)
	*MOD2*	Repression	No activity	Induction	Induction (~100%)
	*FLD*	No activity	Derepr. (~20%)	Induction	Induction (~70%)

Effect and level of derepression compared to the methanol induced level are shown (Induction by methanol for each gene denotes 100%). The data was extracted from the literature given by the citations and are taken from experiments were ammonia was used as nitrogen source.

*Pichia pastoris *is different: at non-growth-limiting conditions both alcohol oxidase genes, *AOX1 *and *AOX2 *are repressed by glucose, ethanol and also by glycerol [61,62,83]. The mRNA level of *AOX1 *upon derepression was ~1–2% of the induced level [92]. The regulation pattern of the *AOX2 *gene was identical to the *AOX1 *gene [93] but *AOX1 *contributes to the vast majority of the expressed alcohol oxidase [62]. Thus knockout of the *AOX1 *gene results in a slow growth phenotype on methanol, called Mut^S ^(methanol utilisation slow), while growth of the *AOX2 *knockout strain on methanol is comparable to the wild type (Mut^+^). Double knockout strains are unable to grow on methanol (Mut^-^). The specific maximum growth rates of the 3 phenotypes are 0.14, 0.04 an 0 h^-1^, respectively [33]. Although glycerol does not lead to derepression of *AOX1 *expression other so-called non-repressing carbon sources like e.g. sorbitol, mannitol, trehalose and alanine do so, but published data do not allow to estimate the strength of the derepression effect [94,95].

*Pichia methanolica*, formerly described as *Pichia pinus *MH4 [96], was one of the latest methylotrophic yeasts developed as an expression host [82]. As earlier described for *P. pastoris*, Raymond *et al*. [82] initially described *MOD1 *(*AUG1*) as the major alcohol oxidase gene and *MOD2 *(*AUG2*) as minor one. Mod1p (Aug1p) was the only alcohol oxidase detected in a methanol-fed bioreactor culture. Also *P. methanolica mod1Δ *(*aug1Δ*) strains displayed a slow growth phenotype on methanol while *mod2Δ *(*aug2Δ*) strains showed wild type growth on methanol. The double knockout strain was unable to grow on methanol [82]. Thus, like with *P. pastoris*, the *MOD1 *promoter serves as a major tool for the expression of heterologous proteins in *P. methanolica*. However, the situation is not that simple. In 1999, Nakagawa *et al*. observed the formation of 9 Mod1p and Mod2p alcohol oxidase hybrid octamers in *P. methanolica *[97]. The fraction of Mod2p in the oligomers was raised with increasing concentrations of methanol (up to 4%) [97]. Induction of *MOD2 *expression by high methanol concentrations is in accordance with earlier findings of K_M _values of MOD homooctamers where MOD-A (corresponding to a Mod1p homooctamer) has a 10-times higher affinity towards methanol (K_M_(MOD-A) = 0.56 mM, K_M_(MOD-B) = 5.6 mM) than MOD-B [98]. It also turned out that the growth phenotypes described by Raymond *et al*. in 1998 [82] was characteristic only for low methanol concentration. As mentioned above, at 1 % methanol [82], the *mod1Δ *strain had the slow growth phenotype whereas the *mod2Δ *strain showed wild type growth. At a concentration of 2 % methanol, both strains grew slowly, although still better than the *mod1Δ *strain at 0.25% methanol. The growth deficiency of the *mod1Δ *variant was still more severe than the one of the *mod2Δ *strain. At 3% methanol both strains grew like the wild type. Under these conditions each of the genes could compensate the lack of the other, thereby restoring wild-type growth rate and biomass yield [99]. In contrast to *AOX1 *in *Pichia pastoris*, *MOD1 *is also expressed by *P. methanolica *when glycerol is used as carbon source, just like the *H. polymorpha MOX *[100,101]. Isolation of ~1.5–1.6 kb long 5' regions of *MOD1 *and *MOD2 *genes as promoter regions and coupling to acid phosphatase as enzyme reporter confirmed previous results from *MOD *isozyme activity measurements: both genes are repressed as long as glucose is present, *MOD1 *is expressed when glycerol is the carbon source. Methanol strongly induced the expression of both, at low concentration mainly *MOD1*, at higher concentrations both genes. *MOD2 *had a regulation pattern similar to the *P. pastoris AOX1 *promoter. It is repressed by glucose and methanol is necessary for induction. No expression is found in glycerol-containing media but, in contrast to *P. pastoris AOX1*, addition of glycerol to methanol medium did not repress *MOD2 *promoter driven expression [101]. Additional regulation of the *MOD1*/*MOD2 *promoters by elevated oxygen concentrations was observed. At low methanol concentrations (0.1%) *MOD1*-driven expression could be boosted by a higher shaking speed where no effect was found for the *MOD2 *promoter. At higher concentrations of methanol (0.3% to 1%) the reverse was true, *MOD2*-driven expression was increased massively while expression driven by the *MOD1 *promoter was unaffected [101]. However, these studies were done in shake flasks with no control of methanol and oxygen concentrations, and the obtained results should have been confirmed with well controlled bioreactor experiments.

The exact physiological role of the *AOX2 *gene in *P. pastoris *is not known. For *P. methanolica *it is assumed to be an advantage under high methanol concentrations. The same was demonstrated in *C. boidinii*. An *aod1Δ *strain which expressed the *PmMOD2 *gene (under the control of the *C. boidinii AOD1 *promoter) exhibited a shorter lag-phase when grown on 3 % methanol, compared to the strain expressing the *Pm*Mod1p or both, *Pm*Mod1p and *Pm*Mod2p. The system using two different subunits with different K_M _values to compose catalytically active hetero-multimers is a belting evolutionary trick for adapting the affinity of the functional alcohol oxidase octamer to the methanol concentration in the medium. The adaptation of subunit composition of the octamer (and therefore the K_M_) to increasing methanol concentrations was mainly regulated by higher expression of the Mod2p subunit under these conditions, as shown by zymogram pattern analyses and reporter studies [99-101]. For *P. pastoris *no studies on the physiological function of the *AOX2 *gene are published which leaves the mystery about its physiological role unanswered.

### Formaldehyde dehydrogenase

Like the alcohol oxidase also the formaldehyde dehydrogenase genes were isolated from all four prominent methylotrophic yeasts. The strength of their promoters, which is comparable to the *AOX *promoters makes them attractive alternatives for recombinant protein production [76,77].

Interestingly, there is an intron at the very 5' end of the gene in *FLD *genes of *C. boidinii*, *P. pastoris *and *P. methanolica*. The size of the intron differs between these 3 yeasts but all are located at the same position, 18 nucleotides downstream of the start codon ATG. No intron is present in the *H. polymorpha FLD *gene. The sequences of the 5' and 3' splice junctions and of the branch points are quite similar if not identical to the respective splice site consensus sequences of *S. cerevisiae *and *S. pombe *but also of higher eukaryotes. After splicing, the 3 genes of *C. boidinii*, *P. pastoris *and *P. methanolica *have the same size as the *H. polymorpha FLD *gene which results in proteins of 380 amino acids length [71,76-78].

Like the other enzymes from the MUT pathway, expression of formaldehyde dehydrogenases is mainly regulated at the transcriptional level [71,78]. In contrast to the alcohol oxidase genes, *FLD *expression is not subject to glucose repression since methylamine or choline are able to induce gene expression even in presence of glucose (as long as ammonia is absent) [71,77,78]. Methanol, formaldehyde, methylamine or choline induced *C. boidinii FLD1 *expression [71]. In *P. methanolica *and *H. polymorpha *the degree of derepression in media lacking glucose differed and was highly dependent on the carbon source. *P. methanolica FLD1 *expression was induced by glycerol, xylose and trehalose although at a much lower level compared to methanol [78]. In the case of *H. polymorpha *xylose, ribose, sorbitol and glycerol were reported to induce *FLD *expression [91]. Addition of methanol further increased the *PmFLD *expression level in presence of glycerol, xylose and trehalose. Glucose does not impede *FLD *expression. Thus, lack of expression is just due to a lack of induction and not due to strong repression. If both glucose and methanol were present in the medium the *FLD *activity level was comparable to the level found with glycerol as sole carbon source. For all these studies ammonia was used as nitrogen source [78]. Interestingly, in *C. boidinii *almost no activity was found with glycerol as carbon source when ammonia was the nitrogen source [71]. If methylamine was used as sole nitrogen source, *FLD *expression was induced in cells even during growth in glucose-containing media and further increased in methanol or glycerol media [78]. Increasing methanol concentrations increased *FLD1 *expression [78], an effect which can also be assumed for *H. polymorpha *where increasing dilution rates in chemostat cultures (which usually means higher steady-state concentrations of methanol) resulted in higher *FLD *activities in the cells [102].

In a *P. pastoris aox1 aox2 *double mutant (KM7121) decreased *FLD *activities were found which suggested a predominant regulation by methanol but also a regulatory contribution by formaldehyde [77]. The same effect was described for *FLD *expression in a *P. methanolica mod1*Δ/*mod2*Δ double knockout strain [78]. *PpFLD1 *activities or *PpFLD1*-promoter driven *lacZ *expression when glucose was the carbon source and methylamine was the nitrogen source reported by Shen *et al*. was not found by Resina *et al*. when expressing *Rhizopus oryzae *lipase under the control of the *FLD1 *promoter [103]. Also no lipase activity was found using glycerol and methylamine as carbon and nitrogen source, respectively. But sorbitol was a non-repressing carbon source since the combination of sorbitol and methylamine resulted in relatively high lipolytic activities [103]. The discrepancies between these two reports can not be clarified since both publications did not show activities in dependence of time nor did they describe exact enough when the cultures were harvested and analysed or if there were residual carbon or nitrogen sources present in the medium. Nonetheless, although some discrepancies appear in reports in the case of *P. pastoris FLD *activity two distinct regulatory systems by the carbon and the nitrogen source are evident. E.g. glucose is not able to repress *FLD *expression when methylamine is the sole nitrogen source and the same is true for ammonia when methanol is used as carbon source. In this respect the regulation in *P. pastoris *needs to be clarified. Thus, we conclude that no repression acts on the *FLD *promoter. No expression is just due to lack of induction. Once either an inductive carbon or nitrogen source is present, both might independently induce expression of the *FLD *gene.

### Formate dehydrogenase

Formate dehydrogenases are frequently used for NADH regeneration in redox biotransformations due to the cheap substrate formate and the gaseous product CO_2_, thereby enabling NADH regeneration without disturbing by-product formation. The most popular *FDH *is derived from *Candida boidinii *[70]. Despite their frequent application, the *FDH *genes are not as well characterised as the two genes described before. So far the *FDH *genes including the respective promoter were only isolated from *C. boidinii *[70] and *H. polymorpha *[104,105]. In the case of *P. pastoris *only a DNA fragment coding for the open reading frame was described [106]. In *C. boidinii*, formate dehydrogenase was not induced by glucose and glycerol. Methanol as sole carbon source or methylamine as sole nitrogen source were inducers of *CbFDH1 *expression. Thus, there is some similarity in regulation to the *FLD *genes. Formate also induced *CbFDH1 *expression, even in the presence of glucose and glycerol. This suggests that formate is a much more powerful inducer than methanol since methanol was just able to induce formate dehydrogenase synthesis in the presence of glycerol but not if glucose was the second carbon source. However, although formate was able to induce synthesis of *Cb*Fdh1p, even in presence of glucose, the induction was much weaker compared to methanol alone or a glycerol/formate mixture as carbon source [70,87]. In *H. polymorpha*, synthesis of formate dehydrogenase is partly derepressed by glycerol. Derepression by xylose, sorbitol and ribose was below 10 % of optimal expression, if present at all [91].

### Dihydroxyacetone synthase

Dihydroxyacetone synthases (*DAS*) and their promoters were isolated from *C. boidinii *[68], *H. polymorpha *[69] and only a *DAS *promoter region was described in *P. pastoris *[92]. Where studied, the *DAS *genes were found to be regulated similarly and as tightly as the respective alcohol oxidase genes. Again, regulation was shown to occur mainly at the transcriptional level [68,69,92]. In general, *DAS *activity was repressed by glucose and induced by methanol. In *C. boidinii *no *DAS *activity was found in media with glycerol as sole carbon source and ammonia as nitrogen source, although at varying extent among the available literature. But addition of methanol resulted in an induction of *DAS *activity up to ~60–70 % of the level obtained by methanol as sole carbon source. Methylamine and choline as sole nitrogen sources also induced *DAS *expression, even in presence of glycerol. Both nitrogen sources failed to induce expression when glucose was the carbon source [68]. Using *S. cerevisiae *acid phosphatase (*ScPHO5*) as reporter enzyme, the *C. boidinii DAS1 *promoter was shown to enable higher expression levels than the *AOD1 *promoter [87]. This result is in agreement with earlier findings in *P. pastoris *where higher levels of β-galactosidase activities were found with a *DAS *promoter than with the *AOX1 *promoter, at least when *lacZ *was employed as a reporter gene [92].

### Catalase

Due to its very important role in disarming the toxic hydrogen peroxide generated by the alcohol oxidase during oxidation of methanol to formaldehyde, catalase (*CAT*) synthesis is also induced when cells are growing on methanol as carbon source. In order to be prepared for the toxic H_2_O_2_, catalase expression precedes the expression of *AOX *and the other genes involved in MUT pathway upon glucose depletion in batch cultures [91,107].

Catalase expression was shown to be controlled mainly on the transcriptional level [86], although some regulation at the posttranslational level, namely import into peroxisomes, has been reported [108].

### Other enzymes involved in methanol utilisation

Several other but less prominent enzymes from methylotrophic yeasts involved in methanol utilisation have been isolated and described. An S-formylglutathione hydrolase gene (*FGH*) was found in *C. boidinii *[73]. The presence of a separate *FGH *enzyme was also reported for *P. pastoris *and *Kloeckera *sp. 2201 (a strain of *C. boidinii*) [72], while in *H. polymorpha FGH *activity was reported to be associated with the formate dehydrogenase [75]. The *C. boidinii FGH *has a typical PTS1-type (peroxisomal targeting signal 1) signal sequence at his C-terminus, in contrast to the *P. pastoris *enzyme. Its localisation in the cell was confirmed via a GFP-fusion to be bimodal. The regulation of expression was similar to *FDH *and *FLD*. Methanol and formaldehyde were able to induce expression and *FGH *is not subject to glucose repression. A *C. boidinii FGH *knockout strain was unable to grow on methanol as sole carbon source in batch cultures, and it also displayed growth defects on methylamine and choline as nitrogen sources when grown in glucose-containing media. Since the *fghΔ *strain was able to grow under methanol-limited chemostat conditions, the authors concluded that *FGH *is not essential but important for optimal growth on methanol. Due to the lower growth rate and growth yield under these conditions, a role in detoxification is assumed for this enzyme [73].

An antioxidant system of *C. boidinii *cells has been described which belongs to the Pmp20 or peroxiredoxin family [109,110]. Expression of this peroxisomal membrane-bound enzyme is highly induced by methanol and absolutely necessary for growth on this carbon source. The *C. boidinii PMP20 *knockout strain did not grow on methanol. Thus the effect of this disruption is more severe than in the catalase knockout strain which is still able to grow on methanol as sole carbon source [109]. Expression of acid phosphatase driven by the *PMP20 *promoter was comparable to the *FDH*-promoter driven expression [87].

Methylformate (MF) was found to accumulate during growth of *C. boidinii *on methanol as carbon source. The enzymatic synthesis of MF was dependent on NAD^+ ^but not dependent on the glutathione-pathway for methanol oxidation [111]. Later on, the enzyme responsible for MF synthesis was described as Zn^2+^-containing alcohol dehydrogenase (*ADH*) [112]. At least three *ADH*-gene products were found to catalyse MF synthesis. From the three genes isolated, *CbADH1 *encodes for the major MFS (methylformate synthase) activity in the cell. An *adh1Δ *strain displayed a prolonged lag phase when grown on methanol, which is even more severe in an *adh1Δ adh2Δ *double knockout strain. Thus, the authors concluded that *MFS *activity plays a major role in formaldehyde detoxification in *C. boidinii *cells growing on methanol as carbon source [113].

### Chemostat studies with methylotrophic yeasts

A large portion of the information on the regulation of MUT pathway enzymes, mainly in *H*.*polymorpha *(CBS4732) and *C. boidinii *(*Kloeckera *sp. 2201), has been published since the late 1970s. For an overview see Table 3.

**Table 3:** Chemostat studies of methylotrophic yeasts [90, 102, 107, 114, 118, 119].

		*AOX*	*CAT*	*FLD*	*FDH*
Methanol-limited chemostat	*C. b*.	Decreasing with increasing dilution rate	Relatively constant over the D range tested	Increasing with increasing dilution rate	Increasing with increasing dilution rate
	*H. p*.	Decreasing with increasing dilution rate	Increasing with increasing dilution rate	Increasing with increasing dilution rate	Increasing with increasing dilution rate

Glucose-limited chemostat	*C. b*.	Low level of derepression at D's < 0.2 h^-1^	Increasing with increasing dilution rate	High level of derepression at D's < 0.4 h^-1^	Low level of derepression at D's < 0.1 h^-1^
	*H. p*.	High level of derepression at D's < 0.4 h^-1^	Increasing with increasing dilution rate	High level of derepression at D's < 0.4 h^-1^	Low level of derepression at D's < 0.2 h^-1^

Glucose/methanol fed chemostat	*C. b*.	Decreasing with increasing D	Increasing with increasing D up to 0.3 h^-1^	Activity peak at D ~0.12 h^-1^	Almost constant at D < 0.3 h^-1^
	*H. p*.	Decreasing with increasing D	Increasing with increasing D up to 0.3 h^-1^	Increasing with increasing D up to 0.3 h^-1^	Increasing with increasing D up to 0.3 h^-1^

Sorbitol-limited chemostat	*H. p*.	Decreasing with increasing dilution rate	Decreasing with increasing dilution rate	Decreasing with increasing dilution rate	Decreasing with increasing dilution rate

Sorbitol/methanol fed chemostat	*H. p*.	Decreasing with increasing dilution rate	Increasing with increasing dilution rate	Increasing with increasing D above 0.3 h^-1^	Activity peak at D = 0.37 h^-1^

Glycerol/methanol fed chemostat	*H. p*.	High specific enzyme activity at D = 0.1 h^-1^	High specific enzyme activity at D = 0.1 h^-1^	High specific enzyme activity at D = 0.1 h^-1^	High specific enzyme activity at D = 0.1 h^-1^

Ethanol/methanol fed chemostat	*H. p*.	No activity found at D = 0.1 h^-1^	No activity found at D = 0.1 h^-1^	No activity found at D = 0.1 h^-1^	No activity found at D = 0.1 h^-1^

The general trend of specific enzyme activity with varying dilution rates (D) are given. *C*. *b*.: *Candida boidinii *(*Kloeckera *sp. 2201), *H*. *p*.: *Hansenula polymorpha *CBS4732

Studies about methanol utilisation enzymes of *H. polymorpha *in a methanol-limited chemostat revealed a clear dependence of specific enzyme activities on the dilution rate. Interestingly, while the specific activities of *FLD *and *FDH *were increasing with increasing dilution rates, or being constant like *CAT *activity, the *AOX *activity decreased significantly [102]. The situation is quite similar in *Kloeckera *sp. 2201 (*C. boidinii*) [107]. Glucose-limited chemostat studies on *H. polymorpha *revealed a remarkable level of derepression of *MOX *activity at dilution rates below 0.4 h^-1^. In *Kloeckera *sp. 2201 *AOX *induction started at lower dilution rates compared to *H. polymorpha*. No induction was detectable above D = 0.2 h^-1^. Below this dilution rate *AOX *activity slowly increased with decreasing dilution rates [114], but always stayed far below the activity determined in a methanol-limited chemostat at the same dilution rates [107]. Almost no alcohol oxidase activity was found in ethanol-limited chemostat cultures at a low dilution rate of 0.1 h^-1^. At this rate significant activity was found in methanol or glycerol-limited chemostat cultures [114], which suggests a much higher repression effect of ethanol compared to glucose. In this context it has to be mentioned that ethanol production was already reported several times to reduce *AOX *promoter driven protein expression [115]. A remarkable result from glucose-limited chemostat cultures was found for *FLD *activities. Both strains exhibited significant derepression of *FLD *activity at D < 0.4 h^-1 ^with an additional boost in the derepression level below D = 0.1 h^-1 ^[114]. At these low dilution rates *FLD *activity reached the level found in methanol-limited chemostat cultures in *H. polymorpha *[102] and 50 % of the *FLD *activity level in *Kloeckera *sp. 2201 at the same growth rate [107].

In chemostat cultivations with a carbon source mixture of glucose and methanol, both substrates were utilised simultaneously to completion by *H. polymorpha *and *Kloeckera *sp. 2201 (*C. boidinii*) at a dilution rate of 0.14 to 0.15 h^-1^, independent of the glucose/methanol ratio of the carbon source. Specific enzyme activities of *AOX*, *CAT*, *FLD *and *FDH *rose with increasing proportions of methanol up to ~20 % (*H. polymorpha*) or 40 % (*Kloeckera *sp. 2201) in the mixture. No further increase in specific enzyme activities was found with higher proportions of methanol in the mixture [116]. On the contrary, the lower the methanol proportion of the carbon mixture, the higher the dilution rate up to which complete methanol utilisation took place [117]. The biomass produced was found to be the sum of the theoretically possible biomass produced from the individual substrates [116]. Thus, both yeast species were able to utilise methanol efficiently in presence of glucose also at dilution rates higher than μ_max _when grown on methanol as sole carbon source. Methanol induction only became possible once a significant level of derepression of *AOX *expression occurred. Specific activity of *AOX *in the chemostat culture with mixed substrates was ~50% of the activity of methanol-limited cultures at the same dilution rate. Similar results were found for the specific activity profiles of *CAT*, *FLD *and *FDH *but, in contrast to *AOX*, activities increased with increasing dilution rates up to ~0.3 h^-1 ^[118]. Increasing specific enzyme activities of *AOX*, *CAT*, *FLD *and *FDH *with decreasing dilution rates were reported for chemostat cultures with sorbitol as sole carbon source. Mixed feed cultures with sorbitol and methanol as a carbon source resulted in a strongly increasing activity of these enzymes starting already at high dilution rates. The specific enzyme activities during mixed feed were comparable to the specific enzyme activities with methanol as sole carbon source at the same dilution rate. A remarkable feature observed with this combination of carbon sources was a peak of formate dehydrogenase activity at a dilution rate of 0.37 h^-1 ^where methanol was only partially utilised [119]. Shift from carbon-limited chemostat cultures with glucose/methanol as a carbon source to nitrogen-limited conditions resulted in severe repression of methanol utilisation genes. Nitrogen limitation, rather than elevated glucose concentrations, were responsible for the repression of MUT pathway genes [120].

Recently, such a detailed chemostat study was published for *P. pastoris*. Identical to *Kloeckera sp*. 2201, *AOX *activity increased with decreasing dilution rates in glycerol-limited chemostat cultures, but this enzyme activity was also just in the range of 1–5 % of the AOX activity found in methanol-limited chemostat cultures at the same dilution rate [121]. The increasing *AOX *activity with decreasing dilution rates on methanol as sole carbon source was almost identical to the trend found in *H. polymorpha *by van Dijken *et al*. [102].

From all these studies it got evident that the degree of sensitivity to carbon catabolite repression by glucose varies between the methylotrophic yeasts studied. Despite the general regulation by methanol, the data also suggest some specific regulation of the individual genes of the MUT pathway since all of them behave differently under varying environmental conditions (e.g. dilution rate, carbon source composition). Varying degrees of derepression or induction for the individual genes as well as for the different host strains under the conditions tested are a remarkable result from these studies. This diversity should be kept in mind when designing a general model for the regulation of the MUT pathway in methylotrophic yeasts.

Although salient differences exist between chemostat cultivation and batch cultivation, results from continuous cultivation under nutrient-limiting conditions for repression/derepression and induction kinetics are in agreement with promoter activities of MUT genes reported for batch-cultures. In contrast to *H. polymorpha*, where a single strain (CBS4732 = ATCC 34438) was used in almost all batch and chemostat studies published, *Kloeckera sp*. 2201 is definitively a different *C. boidinii *strain as the one used for most of the other studies (*C. boidinii *S2). But also in this case, the MUT pathway gene expression data from batch studies and from the chemostat cultivations were in a very good agreement. Unfortunately, *H. polymorpha*, chemostat studies under glycerol-limited conditions were performed only at a dilution rate of 0.1 h^-1^. Since *AOX *expression on glycerol as sole carbon source is the most remarkable difference between the four methylotrophic yeasts studied, comprehensive glycerol-limited chemostat studies of these yeasts will provide interesting insights on the diverse modes of MUT gene regulation.

### *cis*-acting regulatory elements

In addition to gene deletions, batch- and chemostat studies of methylotrophic yeasts molecular deletions within several methanol-inducible promoter sequences from *Pichia pastoris*, *Hansenula polymorpha *(*Pichia angusta*) and *Candida boidinii *were studied. Thereby *cis*-acting elements were identified and some insight into gene regulation was obtained.

#### *HpMOX *promoter

**Figure 2:**
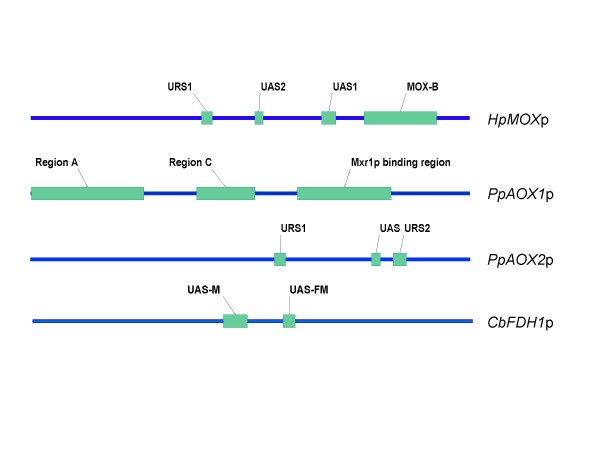
**Regions within methanol utilisation pathway gene promoters identified as *cis*-acting elements: **The regions indicated in this figure were identified either by deletion from the original promoter and proving there activity in a different promoter environment, by footprinting and/or by bandshift assays. *HpMOX*p: *H. polymorpha MOX *promoter [88, 122, 123], *PpAOX1*p: *P. pastoris AOX1 *promoter [125, 126], *PpAOX2*p: *P. pastoris AOX2 *promoter [93], *CbFDH1*p: *C. boidinii FDH1 *promoter [128]; URS1, URS2: upstream repressing sequences, UAS, UAS1, UAS2: upstream activation sequences, UAS-M, UAS-FM: upstream activation sequence for methanol and for methanol and formate, respectively, Region A: region upstream of -690, Region C: region between -540 and -400.

*H. polymorpha MOX *promoter deletions allowed some insight into the regulation of MOX expression [88]. Promoter variants with a spectrum of reporter activity from almost no to 100 % of the wild type were found under derepressing conditions using glycerol as carbon source or under conditions of methanol induction. Three out of a series of regulatory promoter sequence stretches were characterised in more detail (Figure 2): two UAS (upstream activation sequence) and one URS (upstream repressing sequence). First, it should be mentioned, that no significant expression of the reporter protein was found with any of the promoter variants under conditions if glucose was present in the medium. Binding of proteins to the *MOX *promoter within the UAS1/2 and the URS1 region was found by footprinting but neither the attached proteins nor the respective genes were identified. Since the genome sequence of *H. polymorpha *was not known at that time, identification of such proteins should get easier in the near future. The UAS1 binding site was narrowed by the footprinting technique. Its sequence showed a high similarity to a sequence stretch within the co-regulated *CAT *promoter. The UAS2 and URS1 were also identified but no sequence similarity to other co-regulated promoters was described for the latter regulatory promoter elements [88]. Later on, Pereira *et al*. identified a region which is responsible for the repression of the *MOX *promoter in the stationary phase of *H. polymorpha *cultures upon depletion of glucose [122]. The region, called *MOX-B*, is located between the sequences identified by Gödecke *et al*. [88] and the putative TATA box of the *MOX *promoter. Deletion of this region caused a 40-fold increase of reporter enzyme activity in the stationary phase upon glucose depletion, compared to the wild-type promoter at the same conditions. However, this is still not more than 40% of the reporter activity under derepressing conditions in glycerol containing media. The mutant promoter led to the synthesis of ~130 % of reporter enzyme under derepressing conditions [122].

Astonishingly, two *MOX *mRNA species were present during derepression of *MOX *in a *H. polymorpha MOX *multicopy strain. The longer *MOX *transcript (with a minor transcription start at position -425; termed l-*MOX*) was transcribed only in glucose-grown cells. However, no *MOX *activity was observed. Upon induction by methanol, l-*MOX *became down-regulated and transcription of the other *MOX *transcript, originating from the major transcription start point (TSP) at position -25 was highly up-regulated. Only after expression from the -25 TSP, *MOX *activity became apparent. Presence of the l-*MOX *transcript under glucose growth conditions and its repression upon induction were confirmed in the respective *H. polymorpha *wild-type strain [89]. Two transcripts were also observed in *C. boidinii*, although under inducing conditions [63]. Derepression and induction of *MOX *transcription was associated with the repression of l-*MOX*. Upon shift from repressing to derepressing conditions, l-*MOX *transcription was transiently induced followed by a complete inhibition. Therefore a regulatory mechanism involving this TSP shift was postulated [89,123]. Interestingly, it was shown that inhibition of respiration by antimycin A also inhibited this TSP shift [89]. It is thought that respiration is needed for reoxidation of NADH generated during methanol oxidation. Therefore a crosstalk between respiration and methanol metabolism to coordinate gene expression of these two pathways makes intuitively sense. This aspect is further supported by the finding that retrograde signalling from *S. cerevisiae *mitochondria influences peroxisomal gene regulation [124].

#### *PpAOX2 *promoter

Deletion analyses of the *P. pastoris AOX2 *promoter revealed two URS sequences (URS1 and URS2) and an UAS region. Shortening the promoter and adding 1–3 UAS elements to promoter fragments resulted in a strong increase in reporter protein production in the presence of methanol. In shake flask cultures the production of the reporter protein HSA (human serum albumin) could be enhanced up to 120 fold (from 1 to 120 μg ml^-1^) with some of the promoter variants compared to the wild type promoter. The expression was also enhanced 50–60 times just by introduction of a point mutation at position -255 (C to T) or by a duplication mutation between position -233 and -215. Both mutations are positioned within the URS2 element which is located near the TATA box (-204) of the promoter. HSA production levels were also increased in glucose-containing medium, although at a very low level. However, glucose repression could not be overcome in any *AOX2 *promoter construct tested since methanol never induced HSA production in cultures with a mixture of glucose/methanol as carbon source. The minimal HSA levels found in glucose containing media are most probably due to the long incubation time of 72 h, thereby a result of production under derepressing conditions upon glucose starvation. By sequence comparison of this UAS with promoter regions of the *PpAOX1*, *CbAOD1 *and the *HpDAS *genes, homologous sequence stretches were identified but their functions still remain to be elucidated [93]. Although a remarkable increase in *AOX2 *promoter strength was reached, it has to be mentioned that *AOX2 *promoter driven protein expression is usually very low compared to the strong *AOX1 *promoter. By engineering a weaker promoter, it might be relatively easy to reach increased levels compared to a very strong promoter where optimisation hardly ever results in better variants compared to naturally evolved strong promoters.

#### *PpAOX1 *promoter

In the *AOX1 *promoter a region of 243 bp (-415 to – 172) was identified, which is involved in induction by methanol. It has been shown that a homolog of the *S. cerevisiae *transcription factor Adr1p, termed Mxr1p (methanol expression regulator 1) binds to this region [125]. This is consistent with an earlier finding: deletion of ~152 bp within the aforementioned 243 bp resulted in a loss of ~80 % promoter activity under inducing conditions [126,127]. Two further protein binding sites were identified in the promoter region using bandshift assays, one upstream of position -690, and the second between -540 and -400 (note: numbers are counted starting 5' of the ATG start codon of the *AOX1 *gene). A binding event upstream of the -690 position was detectable in medium containing methanol as sole carbon source, but no bandshift was found as long as ethanol was present in the medium. Under both conditions a protein was found to bind between positions -540 and -400, when methanol was the sole carbon source or with a mixture of methanol and ethanol in the medium. Since the two proteins, which are still unknown, do not compete for the two fragments, there is some evidence that they are of different nature [126].

#### *CbFDH1 *promoter

Two *cis*-acting elements were identified by deletion analysis of the *C. boidinii FDH1 *promoter region. Deletion of the first had a strong impact on methanol induction without affecting induction of reporter enzyme (*S. cerevisiae *acid phosphatase *ScPHO5*) expression by formate. This region was termed UAS-M. The second region was involved in both, methanol and formate induction and was therefore named UAS-FM. Induction was carried out with methanol as sole carbon source while formate induction was done with glucose as second carbon source present in the medium. In order to determine the effect of these elements, several promoter variants of the *FDH1 *and the constitutive *ACT1 *(actin) promoter bearing one or more extra copies of the UAS-FM sequence stretch were created. Addition of two extra copies of this element to the *FDH1 *promoter at different positions resulted in a ~2.5-fold increase while addition of four copies resulted in an almost 5-fold increase of reporter enzyme activity upon either methanol or formate induction. The effect was also tested on the constitutive *CbACT1 *promoter. Interestingly, addition of several copies of UAS-FM to the *ACT1 *promoter resulted not only in an inducible promoter, but the effect was even more pronounced than in the *FDH1 *promoter. Addition of two copies resulted in a ~4–5-fold increase of promoter activity while an *ACT1 *variant with four copies of the UAS-FM element displayed an 8–10-fold increase of reporter enzyme activity. While the best *FDH1*-derived promoter was almost 5-times stronger than the wild type promoter, the best *ACT1*-derived variant reached 60 % of the *FDH1 *wild-type strength if induced by methanol. Remarkably, if induced by formate the activity due to the best *ACT1 *promoter variant was ~180% of the *FDH1 *and ~200 % of the *ACT1 *wild-type promoter. Although an induction module was added to the *ACT1 *promoter, no additional glucose repression was found since its activity on glucose was only slightly affected by these modifications [128]. Part of the UAS-FM sequence fits to the consensus sequence of 5 methanol-inducible promoters as defined by Ohi *et al*. [93] and re-defined by Yurimoto *et al*. [87]. These increases in promoter strength are quite remarkable. Unfortunately no further reports were published where these promoters were used for recombinant gene expression.

#### *HpMOX *and *HpFMD *promoter

A rather interesting approach to engineer promoters of the methylotrophic yeast *H. polymorpha *was published by Suckow *et al*. [129,130]. Sequences similar to palindromic sequences of leucine zipper transcription factor (bZip, e.g. Gcn4p, [131]) binding sites were mutated to get a palindromic or a pseudo-palindromic binding site. By introducing palindromic bZip-protein recognition sites by mutagenesis of cryptic binding sites within the methanol-inducible *MOX *and *FMD *promoters and the heat-inducible *TPS1 *promoter in *H. polymorpha*, both an increase in promoter strength was achieved as well as a change in the regulation pattern of these promoters. One engineered *FMD *promoter resulted in an enhanced production of the reporter enzymes β-galactosidase (3.5-fold) and phytase (~1.5-fold) in medium with glycerol as carbon source while a second variant resulted in elevated production rates of the two reporter enzymes (5 and 2-fold, respectively) in glucose-containing media [129,130]. This is a very remarkable result, because enhancing the activity of an already very strong promoter just by introduction of new (pseudo-) palindromic sites (which might act as transcription factor binding sites) might be a very interesting approach in general.

### *Trans*-acting factors and signalling

*H. polymorpha *homologs of *S. cerevisiae *Snf1p and Tup1p, two major players in glucose repression [132], were isolated and disrupted. Although disruption of these proteins had a severe impact on growth of *S. cerevisiae*, no major effect was observed in *H. polymorpha *when grown either on glucose or methanol as carbon source. In contrast to the *TUP1 *knockout where no apparent phenotype could be observed, transcription of the *MOX *and *DAS *genes was reduced under methanol inducing conditions in the *snf1Δ *strain. Interestingly, although the mRNA levels of these two genes were significantly reduced, the growth of the *snf1Δ *was not distinguishable from the wild type strain, indicating that reduction of the *MOX *and *DAS *transcription does not result in a slowdown or bottleneck in methanol utilisation [133].

Expression of *lacZ *under the control of the *H. polymorpha MOX *promoter in *S. cerevisiae *was repressed by glucose. Derepression occurred upon glucose depletion and the observed derepression effect was dependent on the transcription factor Adr1p [122]. Adr1p is a major regulator of the glucose-repressed *ADH2 *gene coding for alcohol dehydrogenase 2 and some peroxisomal proteins in *S. cerevisiae *[134,135]. In contrast to Adr1p, Mig1p (a main repressing DNA-binding protein in *S. cerevisiae *[132]) was not involved in the repression/derepression process of the *MOX *promoter in *S. cerevisiae *[123]. At least in *H. polymorpha *the *MOX *promoter was shown to be organised into nucleosomes [136], and a conformational change in chromatin structure takes place upon derepression [123]. As for several other genes, chromatin remodelling took place during derepression of the *ADH2 *gene in *S. cerevisiae *and this event was presumably a prerequisite for TATA-box accessibility to the transcription machinery and therefore efficient transcription. Adr1p is thought to be involved in this remodelling by triggering chromatin destabilisation thereby enabling transcription upon glucose depletion [137,138].

Two homologs of the *S. cerevisiae SWI/SNF *complex, involved in chromatin remodelling, were isolated and disrupted in *H. polymorpha*. The *H. polymorpha *homologs of the *S. cerevisiae *Snf2p and Swi1p play a role in the regulation of the MUT pathway. Growth of the *H. polymorpha snf2Δ *and *swi1Δ *strains was fully impaired on methanol while they exhibited only a slightly slower growth rate on glucose and sucrose. But in both cases the cultures ultimately reached the same final optical density. On glycerol or oleate as sole carbon source, the strains exhibited an even more pronounced slow-growth phenotype, and the final optical density was also lower. The expression of the native *MOX *and the reporter β-lactamase, both regulated by the *MOX *promoter, were drastically reduced in these mutants (more than 200 fold in the *snf2Δ *and more than 50-fold in the *swi1Δ *strain). Down-regulation of *MOX *might cause the growth defect on methanol [139].

Therefore, a strong evidence exists for an involvement of chromatin remodelling by the *SWI*/*SNF *complex in derepression of the *MOX *and most probably also of the *DAS *promoter in *H. polymorpha*. In *S. cerevisiae *the *SWI*/*SNF *complex was estimated to control ~6 % of all genes, with the control presumably being exerted at the level of individual promoters rather than over chromosomal domains. Therefore, recruitment of the *SWI*/*SNF *complex by DNA-binding regulatory proteins [140] might be a major function of *MOX *binding regulatory proteins in *H. polymorpha *[88,122].

From all the reports published on the regulation of the *HpMOX *promoter, it seems clear that the TSP shift and chromatin remodelling play a major role for the derepression this promoter. Whether these two mechanisms are acting together or just acting in parallel remains to be studied. However, because similar studies are absent for the other methylotrophic yeasts, extrapolation of the results to these yeasts is not possible.

Recently, a key regulator of the MUT pathway and other peroxisomal genes, known as Mxr1p, was identified in *Pichia pastoris *[125]. Binding of this factor to the *AOX1 *promoter sequences was already described in the chapter about *cis *elements. A mutation or deletion in the *MXR1 *gene caused the loss or at least a reduction of transcription of MUT pathway genes and other peroxisomal genes. This was accompanied by phenotypical effects such as a complete loss of the ability to grow on methanol or oleate as carbon source but also a reduction of the growth rates on glucose, glycerol and ethanol [125]. In addition, the deletion caused a complete loss of *AOX1 *promoter driven expression as shown by western analysis and reporter assays using β-lactamase as reporter enzyme. Also the transcription of *DAS *was almost completely abolished. The effects on the other MUT pathway enzymes were not as severe as for *AOX1 *and *DAS*, but still significant. Specific enzyme activities of *FLD*, *CAT *and *FDH *were reduced to ~7 % (*FDH*) or ~20–30% (*FLD *and *CAT*) [125]. In contrast to Adr1p in *S. cerevisiae*, which was regulated on the transcriptional level and expressed only if glucose was absent in the medium [132], Mxr1p was expressed constitutively at a very low level. Its regulation was performed via its subcellular localisation: Mxr1p was mainly in the cytosol as long as glucose was present in the medium and mainly in the nucleus in media containing methanol or oleate as carbon source. Regulation by nuclear localisation is also known for Mig1p in *S. cerevisiae*, however, due to the repressor function of *Sc*Mig1p, its localisation pattern is the opposite of the *Pp*Mxr1p localisation pattern [132].

Interestingly, cytochrome c (*cyt c*), isolated from *P. pastoris *or *S. cerevisiae*, was found to bind *in vitro *specifically to the *AOX2 *UAS found by Ohi *et al*. [93]. *Cyt c *isolated from rabbit heart was not able to bind to this sequence. Mutations within the UAS which were described to abolish the ability to activate transcription *in vivo *also abolished *cyt c *binding *in vitro *[141]. Besides its role in respiration by transferring electrons from complex III to complex IV of the respiratory chain, *cyt c *is also involved in apoptotic pathways after its release from mitochondria (e.g. [142]). Interestingly, employing *AOX1 *promoter based expression, significant fractions of apoptotic cells in deep well plate cultures grown at high glucose or glycerol concentrations led to reduced expression of active enzymes in *Pichia pastoris *[143]. As already discussed in the chapter about *cis*-acting elements, crosstalk of mitochondria and peroxisomes is quite interesting in the context of the high oxygen demand of alcohol oxidase for the oxidation of methanol. As demonstrated and widely discussed in the case of apoptosis, retrograde signalling to the nucleus might also be intuitively logic in the coordination of respiration and peroxisomal function in yeast. Whether cytochrome c is a part of the signalling cascade from mitochondria to the nucleus (where all peroxisomal proteins and most mitochondrial proteins are encoded) or if there is a regulatory role of *cyt c *directly in the nucleus in *P. pastoris *by binding to e.g. the *AOX2 *promoter remains to be elucidated.

A single strand binding (SSB) protein was identified in *P. pastoris *cells [144]. The protein which binds to the near upstream region of the *AOX1 *promoter between -288 and the major transcription start point at -115 was identified as zeta crystalline (*ZTA1*). Interestingly, the SSB activity of recombinant *Pp*Zta1p (produced in *E. coli*) *in vitro *is completely abolished in presence of NADPH while NADH does not have any influence. Although not proven experimentally, the authors speculate on a regulatory function of this protein under oxidative stress conditions as described for the bovine or the *Arabidopsis thaliana ZTA1 *homolog [144].

### Effects of glucose signalling, transport and phosphorylation on gene regulation of MUT pathway gene promoters

Studying glucokinase (GK) and hexokinase (HK) mutants of *H. polymorpha *derived from EMS (ethane methyl sulfonate) mutagenesis revealed the necessity of glucose or fructose phosphorylation for repression by these carbon sources. A hexokinase mutant did not perform fructose repression of *AOX *and *CAT *activities (HK is the only fructose-phosphorylating enzyme) [145,146]. However, since both enzymes (GK and HK) are able to phosphorylate glucose, only the double knockout of GK and HK releases glucose repression. Once no phosphorylation of the substrate occured no repression of the two MUT pathway enzymes studied took place [145-147]. A similar effect was found in *S. cerevisiae *where substrate phosphorylation by either one of the both hexokinases and the glucoskinase was necessary for the repression effect [148,149].

Another *H. polymorpha *mutant insensitive to glucose repression was found to have a mutation in a protein homologous to glucose transporters. The mutant earlier found upon UV mutagenesis (*gcr1-2*) [150] could further be assigned to a gene encoding for a glucose transporter homolog (*GCR1*) [151]. Interestingly, the release of glucose repression phenotype was less severe in the deletion knockout strain compared to the mutated strain. In addition, the mutated strain had no apparent growth phenotype on fructose as carbon source and *AOX *activity was still repressed by fructose. On the contrary, the deletion knockout strain was severely hampered for growth on fructose and repression of *AOX *activity by fructose was released in this strain. This difference was explained by the fact that in the mutated gene, only the transport of glucose was impaired while the function relating to both glucose and fructose was inhibited in the knockout deletion strain [151].

In *H. polymorpha *two glucose uptake systems, one high-affinity and one low-affinity glucose transporter, were described [152]. While the high-affinity transporter was found to be expressed selectively at glucose-derepressing conditions (0.1 % glucose) and when methanol and ethanol are used as carbon source, the low-affinity transporter was found to be acting at high glucose concentrations. The K_m _values for both transporters were estimated to be ~0.05 mM and 1.75 mM, respectively. These numbers were similar to the respective values of the glucose uptake systems found in *P. pinus *MH4 (*P. methanolica*) [152]. Since a *gcr1-2 *mutant strain exhibited a more severe growth defect on low glucose concentrations than at high glucose concentrations, the *GCR1 *gene was thought to encode for a high-affinity glucose transporter [151]. It is noteworthy, that in *K. lactis *the glucose transport system was found to have one major low-affinity glucose transporter, encoded by *RAG1 *and one high-affinity transporter, encoded by *HGT1 *[153]. The unique hexokinase (encoded by *RAG5*), which is the only glucose phosphorylating enzyme in *K. lactis*, was responsible for expression of the transporter genes as well as for glucose repression [154].

Taken together, although part of the work was performed using a different wild type backgrounds, this data depicts a situation similar to that found in *S. cerevisiae *and *K. lactis*. Glucose (or e.g. fructose) uptake and phosphorylation of the substrate by kinases (gluko- or hexokinases) are prerequisites for repression of the MUT pathway. If no substrate sensing or uptake or phosphorylation takes place, the MUT pathway is released from repression.

### Glucose repression mutants and autophagy

Several mutants of the methylotrophic yeasts *C. boidinii *[80], *H. polymorpha *[79,150,155], *P. methanolica *(*P. pinus *MH4) [156,157] and *P. pastoris *[158,159] have been isolated which released the glucose repression of MUT pathway genes. The mutants have high activities of MUT pathway enzymes when grown on glucose or both glucose and methanol as carbon source. When growing on a mixture of glucose and methanol as carbon source, they usually utilise both substrates in parallel, in contrast to the wild type strains which possess a diauxic growth when grown on glucose and methanol mixtures in batch cultures [79,80,150,151]. Mutants with released glucose repression still were repressed by ethanol [79,80,151,157]. Vallini *et al*. isolated several *H. polymorpha *Mut^- ^mutants grouping together in 12 complementation groups. Mutants in only three complementation groups were found to possess very low alcohol oxidase activities due to low promoter strength, not only in methanol, but also in glycerol and methylamine containing medium [160].

Mutants with released glucose repression still showed glucose-induced autophagy of peroxisomes when shifted from methanol to glucose containing medium which reveals again a distinct mechanism for autophagy induction [79,80,151]. Catabolite inactivation of alcohol oxidase and peroxisomes upon shift from methanol to the repressive carbon source glucose was found to be triggered by a short pulse of intracellular cAMP in *H. polymorpha *cells [161]. A cAMP pulse prior to degradation of *AOX *activity was also found in *P. pastoris *when shifted to ethanol-containing media [162]. The mode of autophagy was reported to depend on the intracellular ATP level [163]. The α-subunit of phosphofructokinase was involved in triggering the signal for autophagy in *P. pastoris*. The enzyme, but not the enzymatic activity, was found to be responsible for the onset of peroxisome degradation, indicating a direct role of phosphofructokinase enzyme in signal transduction or different functional domains on the peptide [164].

### Promoters of MUT pathway genes in heterologous hosts

So far, the *P. pastoris AOX1*, *DAS *and *H. polymorpha MOX *promoters were studied in *S. cerevisiae *where they promoted expression of the reporter enzyme β-galactosidase (*lacZ *of *E. coli*) [92,122]. With a few exceptions in the details, the regulation profiles of these promoters in *S. cerevisiae *were similar to their natural hosts: glucose repressed gene expression, during carbon starvation conditions expression was slightly derepressed, and glycerol as carbon source induced expression, even in the case of the *P. pastoris *promoters. β-galactosidase levels expressed under the control of *AOX1 *and *DAS *regulatory regions in *S. cerevisiae *grown on glycerol were comparable to those obtained with the strong *S. cerevisiae CYC1 *and *GAL2 *promoters on raffinose and galactose, respectively [92]. In *S. cerevisiae *expression driven by the *MOX *promoter was also induced by ethanol, methanol or oleic acid. The involvement of Adr1p in derepression/induction of the *MOX *promoter was a very important finding in this context [122].

Two genes coding for the alcohol oxidase isoenzymes, *ZZA1 *and *ZZA2*, are known from a *Pichia pastoris *strain isolated at the UC Davis campus (H. Phaff, strain ID 72–1033). The promoter of *ZZA1 *gene was employed to drive expression of α-amylase in *S. cerevisiae*. In *S. cerevisiae *α-amylase expression from this promoter, which shares 66% identity to the *AOX1 *promoter from *P. pastoris *NRRL Y-11430, was repressed by glucose but induced by glycerol, sucrose and ethanol, which is similar to the *AOX1 *regulation in *S. cerevisiae *[165,166]. Unfortunately, no regulation was described for this promoter in *P. pastoris *therefore no information is available for the regulation of the *ZZA1 *promoter in its own host. Although the regulation pattern is similar in *S. cerevisiae*, no conclusion could be drawn on the similarities or differences between the *ZZA1 *and the *AOX1 *promoter in *P. pastoris*.

As mentioned before, the regulation patterns of the *AOX1 *and the *MOX *genes are significantly different in their natural hosts due to the derepression of *MOX *when glycerol is present. Using the *AOX1 *promoter region in *H. polymorpha *revealed that the this promoter was not repressed by glycerol in the heterologous host [167,168]. For example, expression of the Kunitz protease inhibitor domain of the human β-amyloid precursor protein (KPI) [167] was detected using a glycerol containing medium. Both mRNA as well as protein were found in significant amounts whereas none was detected in a *P. pastoris *expression strain under the same conditions [167]. Similar results were found for the expression of the *S. cerevisiae *invertase (*SUC2*) gene under control of the *P. pastoris AOX1 *promoter in *H. polymorpha *[168]. Thus, the heterologous *AOX1 *promoter is regulated like the homologous *MOX *promoter in *H. polymorpha*. Therefore, the *AOX1 *promoter is well recognised in the heterologous host and repression by glycerol is a host specific effect. Interestingly, the glucose-repressed and maltose-induced *H. polymorpha *maltase gene promoter is also subjected to glucose repression and maltose induction in *S. cerevisiae*, similar to the regulation in the homologous host *H. polymorpha *[169]. Although not a MUT pathway gene, the maltase gene promoter again demonstrates the high degree of conservation of gene regulation between different yeast species.

A very interesting report was published in 2005 about the use of the hypoxia-induced *ADH2 *promoter from *Pichia stipitis *[170] in *Pichia pastoris *for the production of bacterial haemoglobin. Although some control experiments were missing in this paper, such as a comparison to non-inducing conditions and a comparison to a reference promoter from *P. pastoris *(e.g. GAP dehydrogenase promoter), this is another example of a successful transfer of promoters and their functionality in heterologous hosts [171,172].

In the same fashion, the transfer of *S. cerevisiae *promoters to methylotrophic yeasts has been reported. The *ScCUP1 *(*S. cerevisiae *metallothionein, *CUP1*) promoter regulated expression of the *lacZ *gene in *P. pastoris *and consequently resulted in a strong increase of β-galactosidase activity after addition of Cu^2+ ^to the medium. The induction level could be fine-tuned by the Cu^2+^-concentration in the medium in a range between 1 and 200 μM just as in its native host [173]. Thus, these heterologous expression systems offer the flexibility that a researcher can combine a promoter and a host cell to attain a desired expression profile.

## Conclusion

The regulation of the MUT pathway shows similarities among methylotrophic yeasts, but there are some significant differences. In general, all enzymes are induced by methanol and the methanol assimilation pathway enzymes usually are subjected to glucose repression while the dissimilation pathway enzymes are not. However, among the reviewed yeasts there are also significant differences e.g. in regulation of the alcohol oxidase genes: In *C. boidinii *and *H. polymorpha AOX *expression is strongly derepressed by glycerol, although at different levels. Addition of methanol further boosted *HpMOX *promoter activity up to levels reached by induction using methanol as sole carbon source. In presence of glycerol, *AOX *expression is still repressed in *P. pastoris *while *P. methanolica *combines both expression patterns: *MOD1 *is regulated like the *H. polymorpha *and *C. boidinii *alcohol oxidase genes with derepression on glycerol as carbon source while *MOD2 *is regulated similar to the *P. pastoris AOX1/2 *genes where no activity is found in glycerol containing media. *P. pastoris AOX1 *and *AOX2 *are regulated according to the classical repression/induction mechanism which implicates the need for absence of glucose (or glycerol, ethanol) as well as the presence of methanol for full induction of the promoter-driven expression. The *C. boidinii AOD1*, *H. polymorpha MOX *and the *P. methanolica MOD1 *gene regulation might be described better by a repression/derepression mechanism. Absence of the repressive carbon source is sufficient for some degree of expression which differs among the yeasts (see Table 3). Methanol enables full induction of the alcohol oxidase expression also in the presence of the non-repressing carbon source. This is also true for the *P. methanolica MOD2 *gene, although no significant level of derepression was observed using glycerol as carbon source.

The differences found about the regulation of *AOX *promoters among the different yeast species are mainly attributed to the host regulatory machinery and not to the promoter regions. In *H. polymorpha *the *P. pastoris AOX1 *promoter is regulated exactly like the homologous *MOX *promoter. In the *H. polymorpha *cell *PpAOX1 *promoter driven expression is significantly derepressed on glycerol as carbon source. The interesting fact that both regulatory modes are combined in *P. methanolica *raises the question how this yeast is able to distinguish between the two promoters and how the exact regulation takes place. Promoter regulation and glucose repression is highly conserved among yeasts since the glucose-repressed *AOX *promoters are recognised and specifically regulated in heterologous hosts. The promoter brings in the overall regulatory feature like glucose repression and e.g. methanol induction and the yeast supplies the characteristic regulatory pattern caused by different carbon sources, as demonstrated for the *P. pastoris AOX1 *and the *H. polymorpha MOX *promoters. Unfortunately, the puzzling question how *PpAOX1*, *HpMOX*, or *CbAOD1 *driven expression is regulated in *P. methanolica *which combines both regulatory features, is still unanswered.

Despite their very interesting properties, the promoters of the *DAS*, *FLD *and *FDH *genes are not frequently used for recombinant protein production with the exception of the *H. polymorpha FMD *(=*FDH*) promoter, which is as frequently used as the MOX promoter. The *DAS *promoter should be a quite good alternative to the *AOX *promoters since it is as tightly regulated and seems to be even stronger than the respective *AOX *promoter, at least in *P. pastoris *and *C. boidinii *and with the reporter enzymes used so far. *FLD *and *FDH *genes are usually not subjected to glucose repression, thereby enabling induction with nitrogen sources like methylamine or choline in the presence of glucose. In the case of *C. boidinii *the *FDH1 *promoter was also induced with formate in the presence of glucose. Broadening the promoter spectrum in methylotrophic yeasts with different regulatory features will strengthen the position of these yeasts for recombinant protein production and in addition open new challenges and opportunities in metabolic engineering.

In addition, identification of regulatory regions within promoter sequences and the identification of DNA binding proteins and the respective pathways and networks will help to understand the regulation of these promoters. Several transcription factor binding sites were identified in all *AOX *promoters analysed so far. Although several factors involved in glucose repression in methylotrophic yeasts have been identified so far, no complete picture has been generated about the full system of glucose repression and induction by methanol or derepression by other carbon sources works. Aided by extensive available literature, a detailed analysis of gene regulation of the MUT pathway in yeasts should be possible. However, there is just a major problem of comparability of results due to varying reporter enzymes and cultivation conditions. In several cases, the comparison to standard promoter systems is missing, which makes it almost impossible to directly reconcile the data of one publication with another.

Meanwhile the genome sequences of *P. pastoris *and *H. polymorpha *have been determined [174] which should accelerate research on these yeasts. In addition these genome sequences make comparative genomics possible, thereby enabling the transfer of glucose repression mechanism models from the well-characterised yeasts *S. cerevisiae *and *Kluyveromyces lactis *to methylotrophic yeasts and consequently their adaptation.

## Competing interests

The author(s) declare that they have no competing interests.
